# Supplementary material for: Core Endophytic Bacteria and Their Roles in the Coralloid Roots of Cultivated Cycas revoluta (Cycadaceae)
Source: Microorganisms. 2023 Sep 21;11(9):2364. doi: 10.3390/microorganisms11092364 (PMC10537169; doi:10.3390/microorganisms11092364)
Supplement: Supplementary file 1 [file microorganisms-11-02364-s001.zip › Supplementary Figure.pdf]

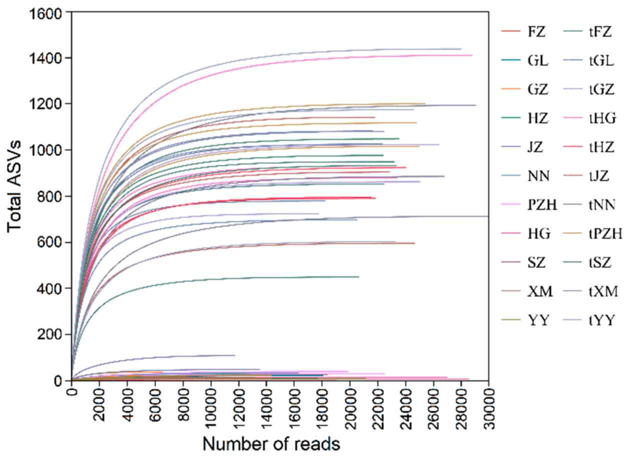

Figure S1: Rarefaction curves of all the 22 samples of coralloid roots and bulk soils of *Cycas revoluta*.

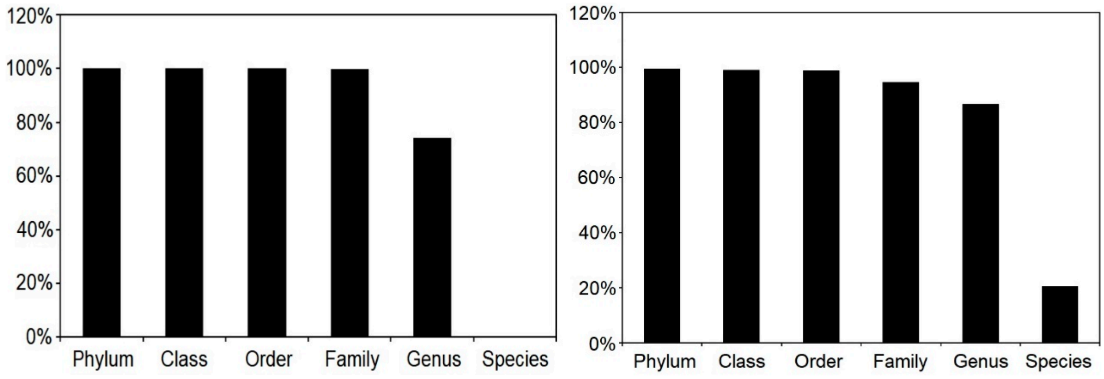

(**a**) (**b**)

Figure S2: Proportion of total bacterial reads assigned to taxonomical ranks. (a) Bacteria in coralloid roots; (b) Bacteria in bulk soils.

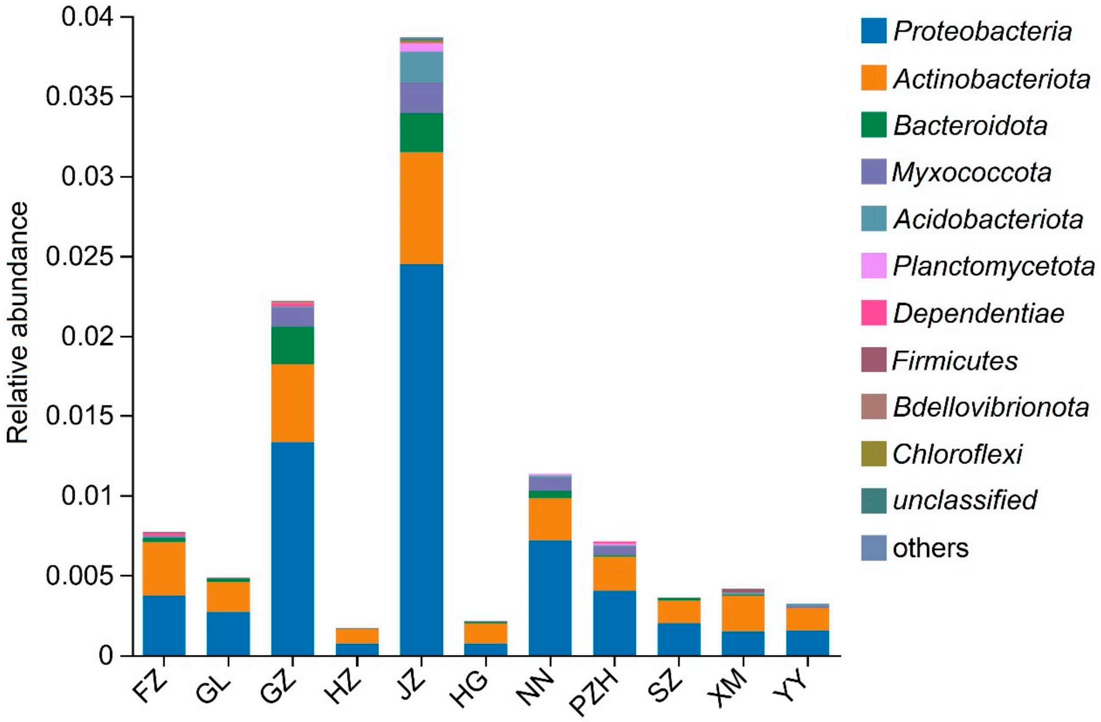

Figure S3: Taxonomic composition of bacterial community without cyanobacteria in coralloid roots of *Cycas revoluta* from different sampling localities. Phyla with relative abundance less than 0.0001% are merged into “others”.

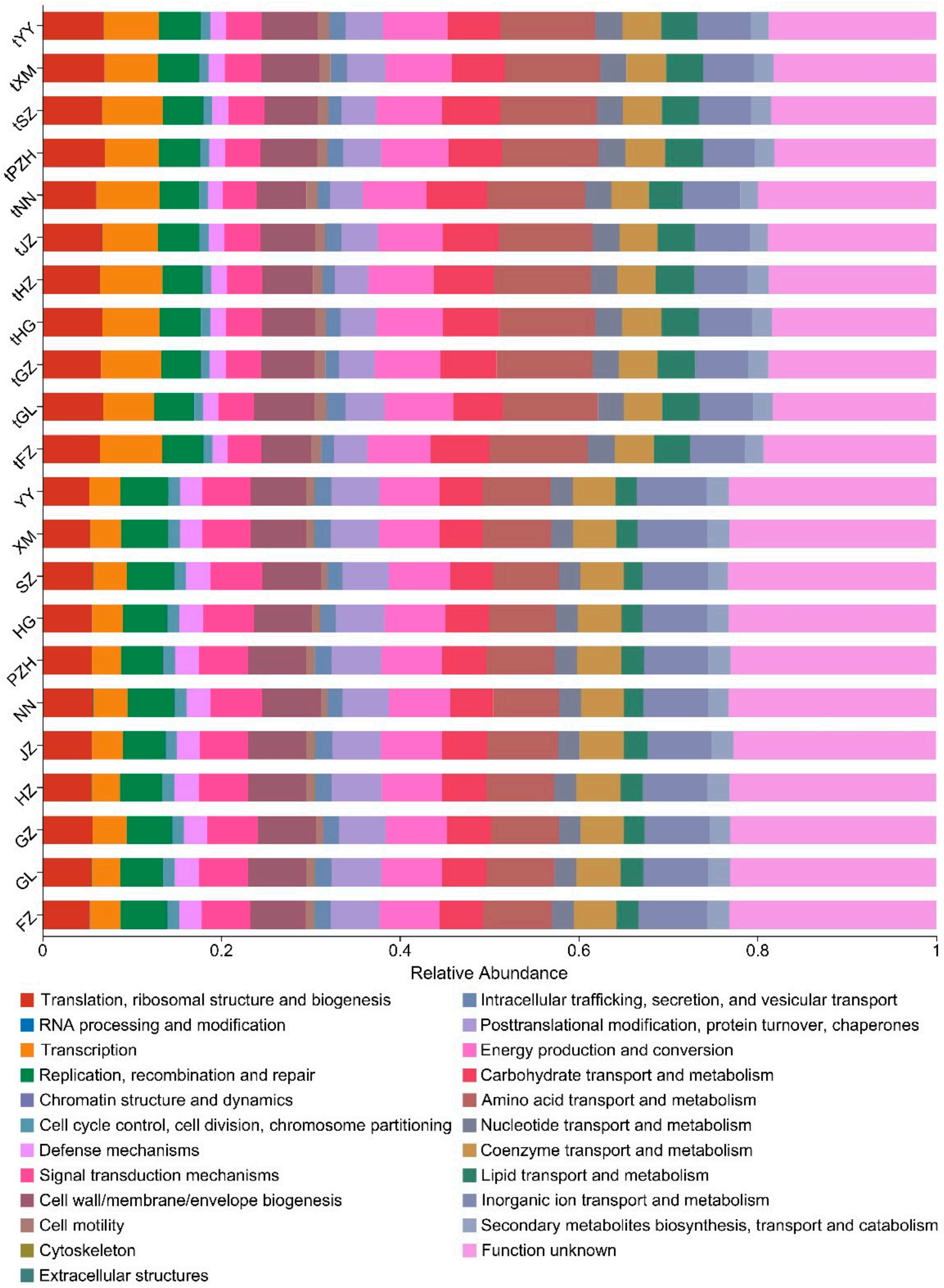

Figure S4: COG functions of bacterial community in coralloid roots and bulk soils detected by the software PICRUSt2.
